# A commentary of “Revealing the mechanism of photoperception regulating glucose metabolism”: Top 10 Scientific Advances of 2023, China

**DOI:** 10.1016/j.fmre.2024.03.008

**Published:** 2024-03-23

**Authors:** Ming Fan

**Affiliations:** a Department of Neurobiology, Beijing Institute of Basic Medical Sciences, Beijing 100850, China; b Chinese Institute for Brain Research, Beijing 102206, China; c Co-Innovation Center of Neuroregeneration, Nantong University, Nantong 226001, China; d Lanzhou University School of Information Science & Engineering, Lanzhou 730000, China

The widespread adoption of artificial light sources, particularly LED lights, catalyzed by industrial modernization, has revolutionized human living and working environments. This transformation, while enhancing life's convenience, harbors potential health implications. Public health studies have identified an association between nighttime light pollution and an increased risk of metabolic diseases such as diabetes and obesity. However, the biological mechanisms through which light regulates glucose metabolism remain unclear.

Professor Xue Tian's research team, through years of comprehensive study, has unveiled the neurophysiological mechanism behind light's regulation of glucose metabolism in biological organisms (mice and humans) [1]. It was discovered in animal models that light signals can be perceived by the intrinsically photosensitive retinal ganglion cells (ipRGCs) in the eye, relayed through the hypothalamic supraoptic nucleus AVP neurons, brainstem solitary nucleus GABA inhibitory neurons, and finally reach the brown adipose tissue via the sympathetic nervous system (retina → hypothalamus → brainstem → brown adipose) (Fig. 1). Light inhibits the sympathetic activity of brown adipose through this multilevel neural circuit, reducing the heat produced by glucose consumption in adipose tissue, leading to a decreased metabolic capacity for glucose. More importantly, Xue and colleagues discovered that a similar mechanism of light perception regulating glucose metabolism exists in humans, with blue light pollution significantly reducing the body's glucose consumption ability. This research not only offers a new perspective on the relationship between light and health but also provides novel approaches for the prevention and treatment of related diseases.

**Fig. 1:**
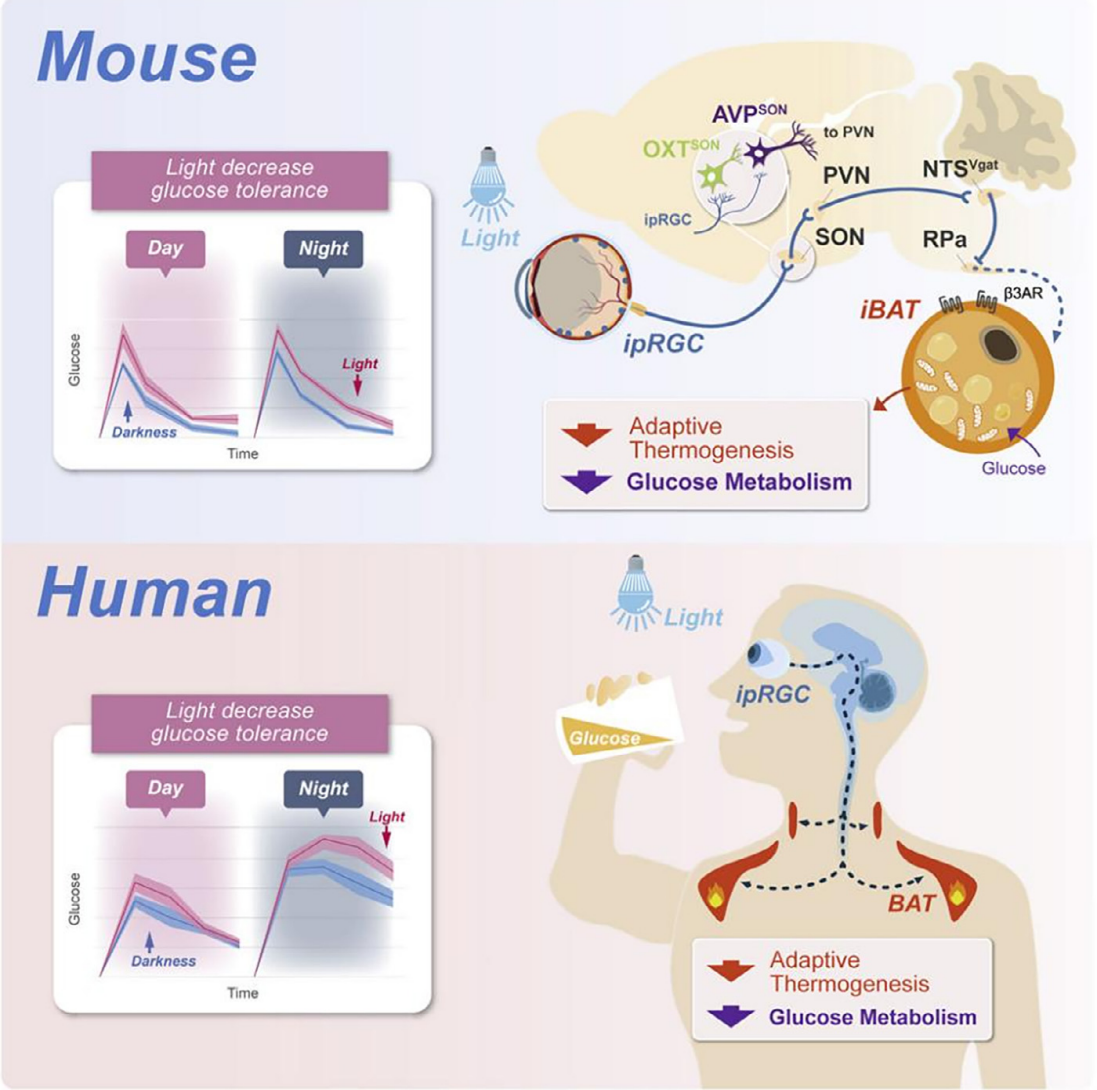
**Light modulates glucose metabolism by a retina-hypothalamus-brown adipose tissue axis**[1].

This study was selected as one of the “Top 10 Scientific Advances of 2023, China” primarily because of its significant achievements in the field of life sciences. The research team was the first to reveal how light influences glucose metabolism via specific neural circuits. This discovery not only addressed a longstanding question perplexing the scientific community but also provided new preventative and intervention methods for the public health sector. The study has enriched human understanding of the relationship between light and health, and may also have profound implications on our lifestyle. By understanding the impact of artificial light sources on human metabolism, we can guide adjustments in indoor lighting design and screen usage habits to reduce health risks. Additionally, this discovery has important implications for scientific research methodology, demonstrating the value of interdisciplinary research and the power of collaboration across fields such as biology, medicine, and engineering.

Here are several future development trends and predictions should be further investigated. **Further scientific questions to address:** Many physiological and pathological processes regulated by light require more in-depth and detailed research. These include exploring the neural mechanisms by which light regulates lipid and protein metabolism; deciphering the plastic regulatory effects of long-term night light exposure on the brain and peripheral metabolic organs; investigating the neural mechanisms through which the brain integrates environmental information (light, stress, etc.) to regulate glucose and lipid metabolism; and studying the impact of extreme light conditions (deep space, deep sea, polar expeditions, tunnels) on organism metabolism. **Application prospects:** Firstly, personalized and intelligent lighting management, with an in-depth understanding of the relationship between light and health, may lead to the development of personalized lighting management systems that adjust lighting conditions based on individual physiological states and health needs. Utilizing ‘Internet of Things’ (IoT) and AI technologies, lighting systems will become more intelligent, automatically adjusting light intensity, color, and timing to protect eye health and improve sleep quality. Secondly, the development of new light sources with spectral characteristics closer to natural light or capable of changing their spectral composition based on environmental needs and demands can minimize adverse effects on humans. Lastly, in terms of public policy on lighting and health, based on scientific research findings, the formulation and enhancement of public health policies and standards pertaining to lighting environments will provide a foundation for the rational use of artificial light sources.

In summary, this research has not only achieved significant academic progress but also carries profound implications for promoting human health, guiding the development of future lighting technologies, and shaping public health policies.

## Declaration of competing interest

The author declares that he has no conflicts of interest in this work.
